# Intravesical drug delivery approaches for improved therapy of urinary bladder diseases

**DOI:** 10.1016/j.ijpx.2021.100100

**Published:** 2021-10-23

**Authors:** Luca Palugan, Matteo Cerea, Micol Cirilli, Saliha Moutaharrik, Alessandra Maroni, Lucia Zema, Alice Melocchi, Marco Uboldi, Ilaria Filippin, Anastasia Foppoli, Andrea Gazzaniga

**Affiliations:** Dipartimento di Scienze Farmaceutiche, Sezione di Tecnologia e Legislazione Farmaceutiche “M.E. Sangalli”, Università degli Studi di Milano, via G. Colombo 71, Milano 20133, Italy

**Keywords:** Bladder, Intravesical delivery, Expandable systems, 3D and 4D printing, Controlled release

## Abstract

Diseases of the urinary bladder have high incidence rates and burden healthcare costs. Their pharmacological treatment involves systemic and local drug administration. The latter is generally accomplished through instillation of liquid formulations and requires repeated or long-term catheterization that is associated with discomfort, inflammation and bacterial infections. Consequently, compliance issues and dropouts are frequently reported. Moreover, instilled drugs are progressively diluted as the urine volume increases and rapidly excreted. When penetration of drugs into the bladder wall is needed, the poor permeability of the urothelium has also to be accounted for. Therefore, much research effort is spent to overcome these hurdles, thereby improving the efficacy of available therapies. Particularly, indwelling delivery systems suited for *i)* insertion into the bladder through the urethra, *ii)* intra-organ retention and prolonged release for the desired time lapse, *iii)* final elimination, either spontaneous or by manual removal, have been proposed to reduce the number of catheterization procedures and reach higher drug levels at the target site. Vesical retention of such devices is allowed by the relevant expansion that can either be triggered from the outside or achieved exploiting elastic and purposely 4D printed shape memory materials. In this article, the main rationales and strategies for improved intravesical delivery are reviewed.

## Graphical abstract

**Figure:**
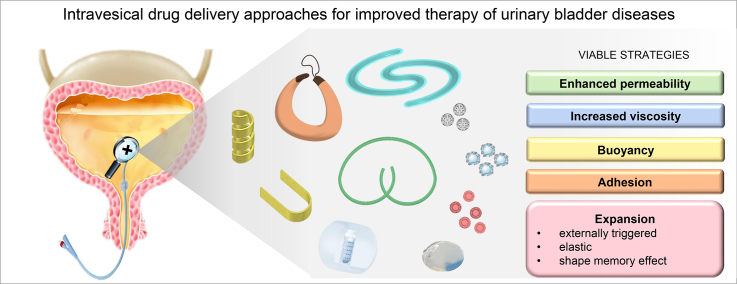

## 1 Introduction

Diseases of the urinary tract, such as incontinence, overactive bladder, interstitial cystitis, bladder cancer and bacterial infections, are widespread in individuals of different ages and gender. However, their incidence increases in elderly people, who represent the population segment of developed countries in continuous growth and whose therapeutic treatments consequently have great impact on healthcare expenses. Particularly, bladder cancer is one of the most diagnosed tumors in the male population worldwide, and is the one with the highest cost from diagnosis to death as well as the fifth most expensive for overall treatment (Leal et al., 2016).

The therapy of the above-mentioned pathologies has mainly involved the systemic administration of drugs by the oral route in some cases coupled with the instillation of liquid formulations directly into the bladder through catheters. Because systemic treatments fail to effectively target the affected tissues, they generally require higher drug doses in order to reach therapeutic concentrations *in situ*. This may lead to overwork of the organs responsible for elimination, which, in the case of elderly people most likely receiving multiple chronic therapies, may increase the risk for complications and onset of side effects. On the other hand, the catheterization procedure used for the local administration of drugs into the bladder is particularly invasive, poorly tolerated and may require to be carried out by specialized personnel often involving repeated hospitalization periods. Furthermore, it has a major impact on the patient quality of life, from a social and relational point of view, causing psychological discomfort, depression, personal disesteem and a sense of loss of control over the bladder function. From an occupational point of view, an increase in the number of absences, not only for therapy administration but also as a consequence of the discomforts arising, is often recorded. As a common side effect, the repeated insertion of catheters for intra-bladder instillation of drugs causes inflammatory phenomena and infections. 10 to 30% of subjects undergoing short-term catheterization develops bacteriuria, often asymptomatic, and after longer-term catheterizations bacteria are found in urine samples from almost all treated subjects. Infections associated with the use of the short-term catheters have been shown to prolong the average hospitalization time from 2.4 up to 4.5 days and have also been correlated with an increase in hospital mortality. Thanks to early detection procedures, in about 75% of patients, bladder cancer is found limited to the mucosa/submucosa area, and this percentage increases when patients under 40 are considered (Babjuk et al., 2019). To reduce mortality and prevent recurrence, the treatment of bladder cancer involves surgical resection and, after surgery, chemotherapy with repeated instillation of anticancer drugs into the bladder. The effectiveness of the current chemotherapy is limited not only by the high risk of dropout of patients due to the problems mentioned above, but also by the difficulties in keeping the active molecules inside the bladder for a sufficiently long period of time and promoting their penetration into the wall. Such difficulties are worsened by the urgency to urinate immediately after installation and by the progressive dilution of the bladder content over time.

The strong therapeutic, social and economic interests related to the vast population affected by diseases of the urinary apparatus have given a decisive boost in recent years towards the identification of drug delivery strategies able to overcome all the issues previously discussed.

The present review aims at presenting a brief *excursus* through the evolution of intravesical administration of drugs focusing on the development of delivery systems capable of maintaining effective concentrations of bioactive molecules within the bladder for strategic periods of time. Indeed, special emphasis was placed on indwelling solid dosage forms for prolonged release, also covering 3D and 4D printed devices.

## 2 Anatomy and physiology of urinary bladder

**Fig. 1:**
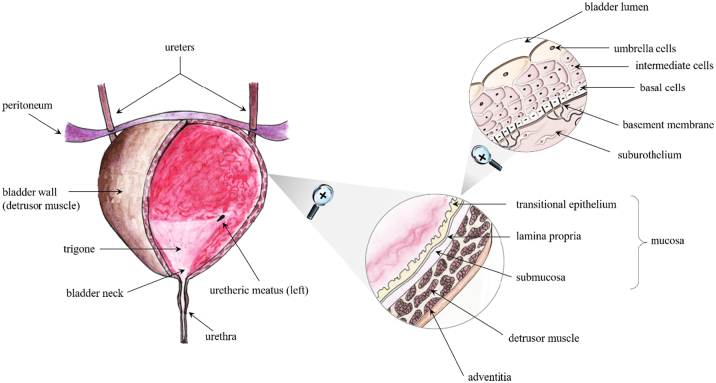
Schematic of urinary bladder. Adapted from (Hamza, 2020).

The urinary bladder is a hollow organ, placed in the anterior part of the pelvic cavity, responsible for the collection and short-term storage of waste substances from the systemic circulation, coming from the kidneys, and their elimination as urinary fluids (Standring, 2005). Its shape, relative position and dimensions vary according to the gender, filling state and condition of the adjacent organs. When empty, the bladder is similar to a tetrahedron, while in the state of maximum filling, under normal conditions around 400–600 mL, tends to assume a pseudospheric shape (Fig. 1).

The fundus, also called body of the bladder, is the portion that constitutes the deposit of urine, where the two ureters coming from the kidneys define a triangular-shaped area together with the internal urethral orifice. The bladder neck, having a length of about 2–3 cm, is connected by the urethra to the external urinary *meatus* that in females also corresponds to the urine exit point, while in males continues into the anterior urethra that runs along the penis.

The bladder wall is made of muscular and epithelial tissues. The mucosa at the interface with the urinary space consists of the uroepithelium, or urothelium, resting on a *lamina propria*. It is a transitional epithelial tissue composed of at least three layers: a basal cell layer attached to a basement membrane, an intermediate layer, and a superficial layer composed of large hexagonal cells (diameters of 25–250 μm) known as “umbrella cells”. The urothelium plays a critical role as a permeability barrier to urine (Khandelwal et al., 2009). Epithelial integrity is maintained through complex processes of migration and proliferation, to restore cell number, and of differentiation to restore function. Basal epithelial cells, which have been suggested to have stem-cell-like properties, typically exhibit very slow (3–6 month) proliferative rates. When the bladder is empty, the urothelium takes on a wrinkled appearance, while it stretches and appears smoother as the amount of urine collected increases. This change is made possible by the ability of the umbrella cells to modify their shape and of the intermediate cells to slide over one another, varying the thickness of the intermediate layer in relation to the state of filling of the organ, without loss of the urothelial barrier function. The bladder muscular layer, which overall constitutes the detrusor muscle, consists in a series of interconnected smooth muscle fibers arranged in different directions: while the fibers belonging to the inner and the external layer mostly show a longitudinal direction, those located in the middle area are characterized by a circular orientation. These three layers converge into the bladder neck and, together with the spiraliform smooth muscle fibers of the urethral sphincter, provide the sphincteric mechanism responsible for urination. Such a process is ruled by a cortical autonomous *stimulus*, which is triggered by the stretching of bladder and urethra walls and causes both the contraction of the muscle tissue here located and the relaxation of the internal urethral sphincter. However, urine release is also under voluntary control, as a voluntary cortical *stimulus* is needed to allow the relaxation of the external urethral sphincter. The presence of approximately 150–200 mL of urine in the bladder initially stimulates urination, which is controlled by myovesical *plexus* sending signals for voiding to detrusor muscle. The latter controls the extent and frequency of bladder emptying.

## 3 Pathology of urinary bladder

Considering the bladder role in the homeostasis of the human body, it is evident how any change in its functionality would necessarily be associated with inconveniences of different severity (GuhaSarkar and Banerjee, 2010; Kolawole et al., 2017; Zacchè et al., 2015). These could be caused either by the natural aging process or by the activation of an inflammatory response, and also be brought about by the onset of various diseases (*e.g.* infections and cancer). Particularly, some chronic vesical pathologies, such as atonic and hyperactive bladder, interstitial cystitis and cancer, are characterized by high current incidence, major discomfort for the patient and limited efficacy as well as tolerability of available treatments. Moreover, such diseases are often followed by opportunistic infections, which can in turn become recurrent.

Atonic and hyperactive bladder, as well as urinary incontinence, relate to an alteration in the control of the muscular activity of bladder wall and sphincters responsible for urination (Chen and Kuo, 2019; Li and Liao, 2016). More in detail, while in the atonic bladder syndrome the contractility of the detrusor muscle is limited and associated with difficulties in performing the emptying process, in the hyperactive bladder disease there is an increased contractility of the organ, thus causing urgency and increased frequency in urination and nycturia. In urinary incontinence, the sphincter control is seriously reduced or lost, with difficulties in, or impossibility of, controlling the urine flow. These diseases are pretty common, showing huge diffusion, and their incidence increases with aging, being slightly higher in men. The treatment generally requires a pharmacological therapy acting on the cholinergic system, chiefly responsible for the modulation of the detrusor muscle tone, on the one hand, and the use of invasive mechanical methods, such as the application of catheters for the correct draining of urine, on the other.

Interstitial cystitis is a painful chronic syndrome with higher incidence in women, mainly associated with a damage in the glycosaminoglycans (GAGs) layer with a loss of vesical epithelium functionality (McLennan, 2014). Symptoms include pelvic pain, urinary frequency, urgency and nycturia. Etiology is linked to alteration of urothelium GAGs, with activation of mast cell, autoimmune reaction and central sensitization. Unfortunately, the therapy is based on empirical studies and suffers from low efficacy. It is limited to the use of a combination of painkillers to be administered systemically and anesthetics instilled *in situ*.

Finally, bladder cancer is one of the most common neoplastic pathologies, the etiology of which lies in the abnormal growth and proliferation of bladder wall cells. Although it generally affects the inner bladder surface (*i.e.* superficial tumors), a small percentage of cases in which tumor cells may infiltrate the muscular layer of the bladder is also described (*i.e.* muscle-invasive cancer). The onset of cancer impacts the normal activity of the bladder, causing hematuria, frequency and urgency in urination together with dysuria. First-line treatment of non-muscle-invasive bladder cancer envisages surgical resection of the tumor followed by local instillations, repeated over time, of different chemotherapeutic agents (*e.g.* epirubicin, mitomycin, Bacillus Calmette-Guérin) to prevent recurrence and progress into muscle-invasive bladder cancer (Babjuk et al., 2019; Yoon et al., 2020).

## 4 Administration of drugs into the bladder and relevant open issues

Local treatment of bladder diseases, despite being a poorly acceptable choice for the patient, exhibits the advantage of reducing the side effects of specific drugs. Indeed, substances that would not be considered safe when administered *via* other routes, such as dimethyl sulfoxide and botulinum toxin, have been approved for intravesical infusion. Moreover, compared to the oral route, partial elimination due to the first-pass effect can be avoided, thus allowing possibly lower drug strengths to be used. Local administration is made by instilling liquid formulations containing one or more drugs into the bladder cavity, through a catheter inserted directly into the urethra of the patient; such liquids can be left *in situ* for a pre-determined time lapse before being excreted or withdrawn.

The use of catheters is a widespread practice, not only exploited to support the treatment of urinary system diseases, but also for diagnostic purposes. However, catheterization causes inconvenience and discomfort to the patient, which is generally treated by local application of ointments or gels containing anesthetic drugs having short-lasting efficacy (usually limited to minutes).

**Fig. 2:**
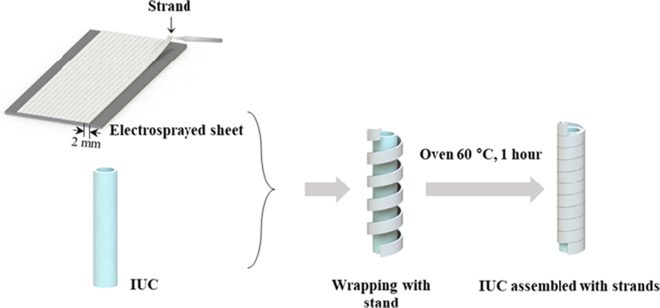
Schematic of the fabrication procedure of strand-wrapped indwelling urethral catheters for lidocaine release. Reprinted with permission from (Kim et al., 2021).

Indwelling urethral catheters (IUCs), also named Foley catheters, are designed to remain in place for many days or weeks and to be held in position by an inflated balloon in the bladder. They are typically used to enable urinary bladder emptying in the case of pathologies impairing natural urination or to drain urine when the patient is bedridden. The tube of the catheter has two separated lumens, or channels, running down its length. One lumen, open at both ends, drains urine into a collecting bag; the other has a valve on the outer end and connects to the balloon at the inside tip. The balloon is inserted through the urethra and positioned deflated inside the bladder. Then it is inflated with a prefixed volume of sterile water to maintain the position and avoid accidental slipping out. For removal, the balloon is deflated, and the catheter is simply pulled out. Prolonged release of an anesthetic for reducing the pain induced by IUC has been recently proposed by Kim and co-authors, who designed a drug-loaded polymer strand releasing lidocaine up to 7 days (Kim et al., 2021). The thin poly(lactic-*co*-glycolic acid) (PLGA) strand was wrapped around the external tube of the urinary catheter and released the drug from the surface thus alleviating the discomfort associated with the presence of the device (Fig. 2).

When urinary catheters are placed in the bladder through the urethra for days or weeks, long-term residence often entails other complications such as the onset of infections. These are generally caused by bacteria found in the external area close to the urinary orifice, such as *Escherichia coli*, *Enteroccoccus fecalis, Proteus mirabilis* and *Pseudomonas aeruginosa*, which could reach the bladder either during catheter insertion or by moving upstream through the catheter cavity from the drainage bag where they can proliferate. Once in the bladder, such microorganisms form complex structures called biofilms that make penetration of drugs, and thus the relevant action, even more difficult. In any case, the elimination of pathogens is fundamental to avoid worsening of infections and further problems, and it generally requires systemic and local administration of antibiotics.

Several papers reported on the coating of urinary catheters with drug-containing formulations in order to reduce such a risk. For instance, salicylic acid-releasing polyurethane acrylate polymers, silver nanoparticles, antibacterial polycationic nanospheres and chlorhexidine-loaded poly(ethylene glycol)-*block*-poly(ε-caprolactone) micelles were investigated (Francesko et al., 2016; Gefter et al., 2018; Nowatzki et al., 2012; Srisang and Nasongkla, 2019; Thomas et al., 2015).

A further issue involved in therapies based on intravesical administration of drugs, particularly those for bladder cancer, is given by the poor permeability of urothelium, which may represent a tough barrier to be crossed especially by molecules with high molecular weight (Yoon et al., 2020).

In addition to the problems associated with the poor urothelium permeability, it should be considered that the maximum residence time of drugs instilled into the urinary bladder is approximately of 2 h and their concentration is constantly diluted by excreted liquids drained by ureters, with consequent need for frequent instillations to maintain the desired therapeutic properties. Even though drug elimination after the instillation treatment can be deferred by restricting the liquid intake, which reduces the production of urinary fluid, and urinating before intravesical infusion, compliance issues may arise especially when elderly patients are involved, for whom holding the urge to urinate and complete bladder emptying are often problematic. Moreover, the large variability in the residence of drugs *in situ* makes it difficult to define personalized therapeutic regimens for individual patients, thus implying the application of rigid therapeutic protocols.

For all of these reasons, the distress caused by catheterization and discomfort of frequent treatment regimens are connected with high dropout rates, with severe consequences especially in the case of bladder cancer.

## 5 Strategies to improve the pharmacological therapy of urinary bladder diseases

Based on the above considerations, it is clear that the local therapy of urinary bladder diseases is a challenging goal. The success of such treatments necessarily depends on proper exposure of the organ to the drug (Wang et al., 2021). Therefore, strategies to improve the outcome of intravesical pharmacological treatments have been aimed at maintaining effective drug levels *in situ* through the use of delivery systems able to be retained and release the drug into the organ for a prolonged period of time and/or enhancing permeation of locally administered drugs throughout the bladder wall.

### 5.1 Enhancement of urothelium permeation

**Fig. 3:**
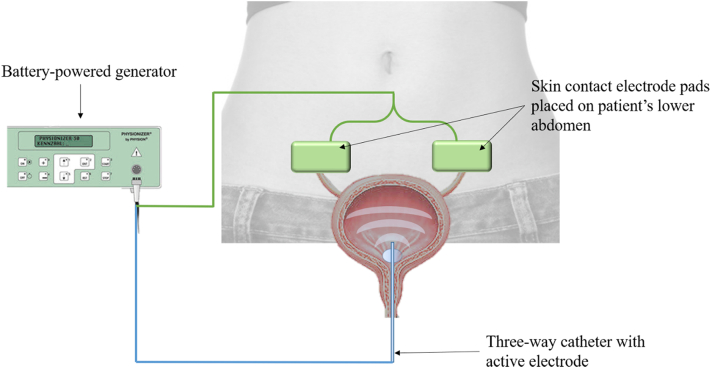
Schematic of intravesical device-assisted therapy for non-muscle-invasive bladder cancer electromotive drug administration.

Enhancement of permeation of the urothelium has been obtained through intravesical device-assisted therapies. Radiofrequency-induced thermochemotherapeutic effect (RITE), conductive hyperthermic chemotherapy, and electromotive drug administration (EMDA) have shown promising results (Tan and Kelly, 2018). In particular, EMDA is based on the application of electric impulses through a catheter equipped with an inner electrode (Fig. 3). Its use for the treatment of non-muscle-invasive bladder cancer demonstrated enhanced transmembraneous transport of mitomycin-C into all bladder wall layers compared to passive diffusion following standard instillation (Di Stasi and Riedl, 2009).

Nanocarrier drug delivery systems, formulated from lipids, polymers, proteins and metals, have also been leveraged to increase the penetration across the bladder mucosa (Zacchè et al., 2015). In particular, liposomes are based on concentric bilayers with nanometric sizes and composition mimicking the human cell membrane. Liposomes for intravesical administration of lipophilic or hydrophilic drugs have been described as well as solid lipid nanoparticles, nanoparticles with specific ligands for cell targeting, silver, gold or magnetic nanoparticles, and branched polymeric dendrimers (Fraser et al., 2003; GuhaSarkar and Banerjee, 2010; Sansare et al., 2021; Tyagi et al., 2016; Yu et al., 2020). Peptide molecules having less than 40 amino acids showed an intrinsic capacity of transduction across biological membranes and have been exploited to transport various substrates inside cells. Drug conjugates with arginine-rich peptides have been described to enhance intracellular uptake of the active molecule (Nakase et al., 2017). Specific peptides have also been used in liposome, nanoparticle and microparticle formulations showing effectiveness in promoting drug permeation *in vitro* and *in vivo* (Hsieh et al., 2011). Chemical agents, such as polymers, dimethyl sulfoxide and protamine sulphate, have been also proposed as enhancers (Chen et al., 2003).

However, both EMDA and substances employed to promote permeation may induce irreversible alteration of urothelial cells, interfering with the relevant barrier function and causing unwanted side effects.

### 5.2 Prolongation of vesical residence time

A common strategy to extend the contact time of intravesical formulations with the luminal surface of the bladder is based on increased adhesivity or viscosity of solutions or suspensions instilled by catheterization (Kolawole et al., 2017). Mucoadhesive systems are based on polymers able to interact with the urothelial GAGs (Chatta et al., 2015). As viscosity enhancers, thermo-sensitive polymers, which present low viscosity at low temperature (refrigeration temperature) and undergo rapid gelation at higher temperature (*e.g.* TCGel®), are used (GuhaSarkar et al., 2017; Qiu et al., 2020). Solutions/suspensions containing such polymers can easily be instilled into the bladder where the body temperature activates the formation of a gel depot, having adhesive properties, from which the drug could be slowly released *via* diffusion/erosion mechanisms. Viscosity of liquid formulations can also be increased by polymers sensitive to ionic concentration changes. This may be relied on to have originally syringeable formulations thickened in the bladder environment due to the presence of ions in the urine triggering the relevant gelation (Vigani et al., 2020). Even though *in situ* gel formation is a good option for prolonging intravesical residence time, the risk of urethra obstruction due to the increased viscosity of urine might be quite problematic. Moreover, adhesion of gels to the luminal surface of the bladder can affect the urothelium structure inducing inflammatory reaction.

To prolong residence time in the bladder, floating systems have also been proposed (Kolawole et al., 2017; Lin et al., 2014a, Lin et al., 2014b; Zhu et al., 2016). These exploit buoyancy of low-density dosage forms in urine, thus resisting excretion through the urethra. The approach is based on the use of excipients generating CO_2_ when in contact with aqueous fluids as occurs with effervescent formulations. Besides NaHCO_3_ or NH_4_HCO_3_, perfluoropentane (PFP) has been used as a solid material that converts to gas when subjected to temperatures above 29.2 °C (Zhu et al., 2016). Despite the promising approach, floating systems have been poorly investigated, and the risk of occlusion of the urethra should be accounted for when the urine volume in the bladder is low. Excessive gas production could also induce expansion of the organ wall with consequent need for urination.

#### 5.2.1 Expandable devices for prolonged drug delivery

Indwelling drug delivery devices are physical systems administered by transurethral catheterization aimed at prolonged release of active pharmaceutical ingredients in the urinary bladder. Upon insertion into the bladder, they undergo an increase in spatial encumbrance when the bladder neck is passed, which enables intra-organ retention. As with other intravesical delivery systems previously discussed, it is important that physiological urination is not hampered by the device. Indwelling systems can be water-soluble or biodegradable and therefore designed to be excreted spontaneously by urination: at the end of the drug release process, they may dissolve or release fragments able to pass the urethral sphincter freely. These portions should be small enough to avoid urinary tract obstruction. On the other hand, insoluble and/or non-degradable intravesical devices require removal procedures after depletion. Potentially, indwelling vesical systems may extend intra-organ residence and related drug delivery over time periods in the order of few to several days.

The mechanism allowing for increase in spatial encumbrance may rely on external triggering of expansion, elastic relaxation or shape memory effect. In Table 1, an overview of the main expandable devices reported in the literature is presented.

**Table 1:** Expandable devices for prolonged drug delivery.

Schematic of the device	Expansion mode	References
UROS infusor	Drug reservoir with a pressure responsive-valve. Expansion is achieved after filling with the drug solution	(Situs Co, 2000)
		(Matsuura et al., 2001)
Intravesical balloon	Intravesical balloon inflated with drug formulations and positioned within the bladder *via* magnetic control	(Innoventions Ltd, 2000)
		(Yachia and Hirszowicz, 2001
		(Yachia and Hirszowicz, 2002)
		(Yachia and Hirszowicz, 2006a, Yachia and Hirszowicz, 2006b)
Multiple spherical units device	Drug-containing polydimethylsiloxane microspheres embedded in biodegradable matrix units that are connected by flexible resorbable suture threads. The retentive configuration is achieved by pulling the threads	(Hopmann et al., 2015)
S-shaped 3D printed hollow device	Drug reservoir fabricated by SLA 3D printing based on an elastomer. The retentive configuration is achieved after catheter removal	(Xu et al., 2021)
LiRIS™ and GemRIS™	Silicon tube prefilled with the drug formulation. The retentive pretzel-like configuration is achieved thanks to the superelastic properties of a nitinol wire that regains its starting shape after catheter removal	(Lee et al., 2007) (Nickel et al., 2012)
		(Giesing et al., 2015)
		(Lee and Daniel, 2015)
		(Cima and Lee, 2020)
Osmotic device based on elastomeric materials	Biodegradable elastomer-based device having osmotic release mechanism	(Tobias et al., 2010)
PVA-based 4D printed intravesical device	U- and helix-shaped PVA matrices, fabricated by HME and 4D printing *via* FDM and deformed to an elongated temporary shape for insertion into the bladder *via* catheter. Retentive configurations achieved thanks to shape memory effect induced by exposure to urine at body temperature.	(Melocchi et al., 2019a)

##### 5.2.1.1 Devices based on externally-triggered expansion

An intravesical system resembling indwelling urethral catheters is the UROS oxybutynin infusion pump by Situs Corporation (Oxybutynin Intravesical — Situs, 2002). This includes a reservoir that can be easily inserted empty into the bladder and filled from the outside with the desired drug formulation (Matsuura et al., 2001). The reservoir, made of poly(dimethylsiloxane), is large enough not to be drained out even when voided but not so large as to cause bladder irritation or occlusion (dimensions before filling: 100 mm in length and 6 mm in diameter). After the device is filled, it is allowed to float freely or alternatively is tied to the bladder wall. The two ends are connected with an inextensible material (polyester ribbon), which allows annular shaping. In order to deliver the drug at a controlled rate, the device is equipped with a pressure-responsive valve. The flow resistance of the latter is sensitive to the pressure at which the drug is stored inside the chamber. The UROS system is proposed for delivery of drugs for approximately 30 days, after which it is removed by urethral cystoscopy (Fraser et al., 2002; Cima et al., 2014). Tested *in vivo* in healthy volunteers, it provided clinical benefits in terms of reduced frequency and urgency of urination (Situs Co, 2000). Nevertheless, it failed to go beyond Phase II.

Yachia and Hirszowicz patented an invention for intravesical drug delivery with the aim of treating urinary incontinence, chronic urinary infections, cancer or of monitoring the bladder activity (Innoventions Ltd, 2000; Yachia and Hirszowicz, 2001). The system is based on an expandable balloon which can be inserted into the urinary bladder and filled afterwards, or filled and compressed prior to insertion. A magnetic element located at the inner surface of the balloon or embedded in its wall can help the relevant positioning for sealing the bladder or directing to specific regions of the urothelium. In addition, the device has a self-sealing valve in the wall of the balloon, which prevents the fluid from leaking out after the needle used for filling is withdrawn. The device may float or sink into the urinary fluid depending on the type of filling.

Another system for intravesical drug delivery has been proposed by Hopmann and co-authors for the treatment of overactive bladder syndrome (OAB) (Hopmann et al., 2015). The device is composed of multiple spherical units having a diameter of 2.4 or 4 mm connected by a flexible and resorbable suture thread. The spherical units consist of foamed matrices of poly-D, l-lactide-*co*-glycolide-co-polyethylene glycol diblock copolymer (PLGA-PEG) embedding microspheres of poly(dimethylsiloxane) loaded with trospium chloride, an anticholinergic drug. The device is arranged in a way that it can be expanded in the bladder after insertion through the urethra *via* a catheter. The retention mechanism is activated externally by pulling the threads, and the change in shape allows the system to safely be maintained inside the bladder. After degradation of the PLGA-PEG matrix, the microspheres are eliminated in the urine. The approach relies on continuous release of the drug from the microspheres and complete elimination of device residues after less than 4 weeks, to avoid the onset of potential side effects such as the appearance of scale due to the deposit of salts on the microparticles.

##### 5.2.1.2 Systems based on elastic materials

Elastic materials are able to regain their shape following the removal of an external force (*i.e.* compression, bending, stretching). Polymers presenting these features are named elastomers. They express weak interchain forces, low Young's *moduli* and are characterized by high values of failure strains. Mechanical properties of elastomers are particularly advantageous for numerous pharmaceutical applications and are ideal for devices that need to acquire different shapes for insertion and retention in hollow organs (Lendlein and Langer, 2002). However, one of the major drawbacks of intravesical devices based on elastic materials is the need for a transurethral removal procedure, which unavoidably reduces the patient compliance.

**Fig. 4:**
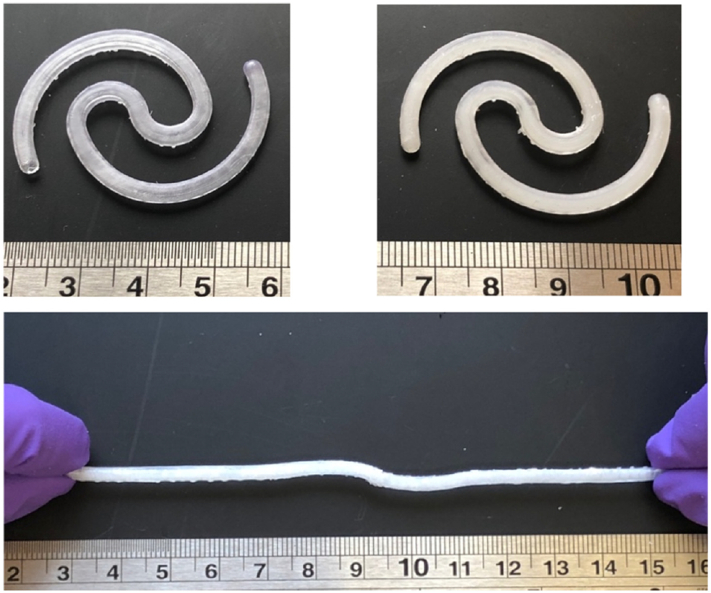
Photograph of the SLA 3D printed hollow device before (top left) and after (top right) filling, and under stretching (bottom). Scale in cm. Reprinted with permission from (Xu et al., 2021).

Recently, an elastomer-based system for intravesical delivery was prepared by 3D printing using stereolithography (SLA) (Fig. 4) (Xu et al., 2021). SLA consists in the polymerization of liquid monomers by light irradiation for obtaining precise and complex 3D geometries, smooth finish and high resolution (approximately 25 μm). The device composition includes a thermosetting resin that is polymerized by 405 nm laser light. Lidocaine hydrochloride has been added to the liquid resin prior to printing, thus being uniformly distributed in the printed item, or filled in after printing of hollow shells. Different shapes (solid and hollow) and drug loadings have been evaluated for mechanical properties that should allow the device to fast regain its starting shape after deformation by stretching. The systems offer satisfactory mechanical characteristics and the desired tuneable release behavior.

**Fig. 5:**
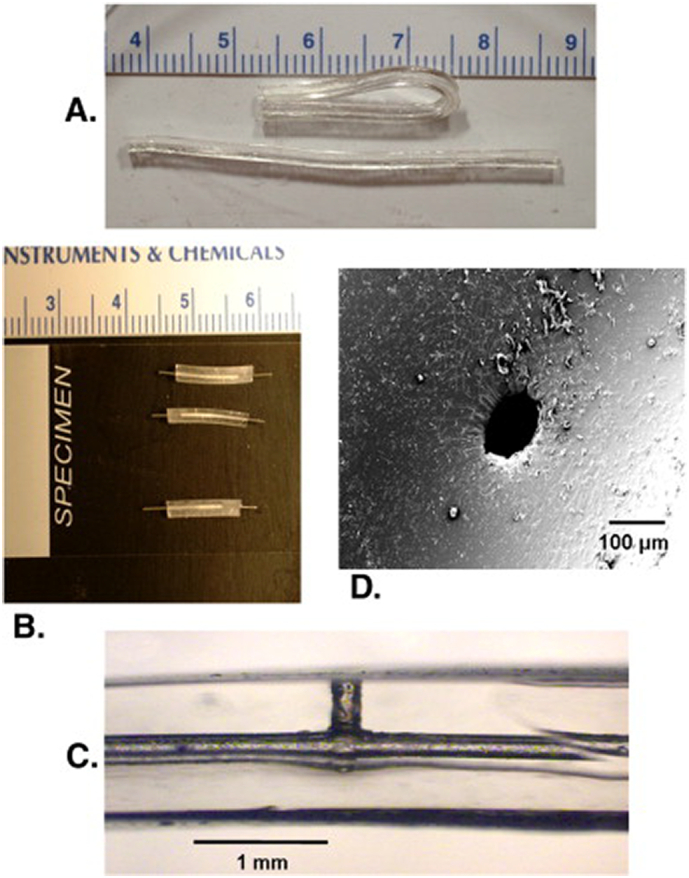
Photographs of the reservoir system based on a biodegradable elastomeric polymer. A) tubes demonstrating flexibility of material. B) modules loaded with drug and plugged with steel wires. C) zoomed-in view of module having 150 μm diameter laser-drilled orifice channel leading into 300 μm diameter core. D) SEM image of 100 μm diameter laser-drilled orifice viewed from the orifice surface. Reprinted with permission from (Tobias et al., 2010).

A reservoir ciprofloxacin hydrochloride system prepared from a biodegradable elastomer has been proposed for urinary bladder and, more in general, local urological therapies (Tobias et al., 2010). The device is composed of poly(glycerol-*co*-sebacic acid) casted in a tubular geometry and filled with drug powder (Fig. 5). A laser-drilled orifice allowed the release of the drug induced by osmotically-driven water permeation. *In vitro* experiments have demonstrated that the elastomer is susceptible to hydrolytic degradation indicating the possibility of producing a completely resorbable drug delivery device. In addition, modulation of the release rate is achieved by varying the orifice size.

**Fig. 6:**
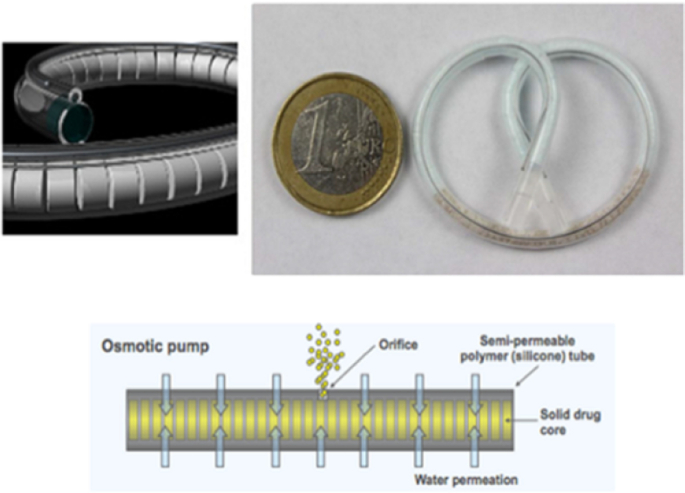
Images of GemRIS™ (top) and schematic of the device in operation (bottom). Reprinted with permission from (Grimberg et al., 2020).

TARIS Biomedical is a pharmaceutical company with extensive experience in intravesical drug delivery. Among various devices proposed over the years, GemRIS^ΤΜ^ (gemcitabine-releasing intravesical system) and LiRIS^ΤΜ^ (lidocaine-releasing intravesical system) have been developed taking advantage of the superelastic characteristics of nitinol (Cima and Lee, 2020; Daneshmand et al., 2017; Grimberg et al., 2020). This material, an almost equiatomic metal alloy of nickel and titanium, exhibits not only superelasticity (good combination of high strength and low elastic module) but also high corrosion resistance, non-ferromagnetic behavior, biocompatibility and, importantly, shape memory, unique properties that are largely exploited in the area of medical devices (Fig. 6). GemRIS^ΤΜ^ and LiRIS^ΤΜ^ show a common design but differ for the drug conveyed. Both are conceived as an osmotic pump, leading to a prolonged release for approximately 2 weeks. The final goal is to reduce as much as possible the overall dimensions of the system with respect to those already available (*e.g.* UROS) in order to limit patient discomfort. The reduction in size without decreasing the payload has been attained because the drug is present in the solid state rather than as a solution. The devices include a water-permeable silicone tube with different inner cavities. While the larger cavity is filled with mini-tablets containing either lidocaine hydrochloride or gemcitabine, the smaller one contains a nitinol wire. The pretzel-like shape of the systems prevents emptying from the bladder. To enable intravesical administration, the wire is mechanically forced into an elongated shape. This way, it is possible to insert the system into a catheter, which acts as an external constraint. When the catheter reaches the bladder and the DDS is positioned into the cavity, nitinol regains the initial pretzel-like shape thanks to its superelastic behavior, thus promoting retention in the target area.

In a phase 1 study, the systems have been well tolerated while retained in the bladder in both volunteers and interstitial cystitis/bladder pain syndrome patients, even when administered to healthy subjects in a *placebo* form (Nickel et al., 2012). In the case of LiRIS^ΤΜ^, this rules out any possible misleading effects on tolerability caused by the conveyed anesthetic drug. Pain relief and reduction in voiding urgency and frequency have been reported after a two-week treatment together with signs of bladder healing. Two phase 2 studies have also been carried out, in which no significant treatment effect was assessed with lidocaine hydrochloride 400 mg LiRIS^ΤΜ^ as compared with *placebo* (Evans et al., 2021).

However, one of the major drawbacks of the described devices based on elastic materials is the need for a transurethral removal procedure, which unavoidably reduces the patient compliance.

##### 5.2.1.3 Devices based on shape-memory materials

Shape memory polymers (SMPs) are able to undergo major shape modifications under the application of a range of triggering *stimuli*, such as changes in temperature, light, magnetic field and electrical currents, which could even be applied remotely (Lendlein and Langer, 2002; Melocchi et al., 2021c; Shin et al., 2017; Zhang et al., 2019). The rising interest towards these polymers could be considered a consequence of the spread of shape memory alloys (SMAs), which dates back to the end of the 90s. In this respect, among SMAs, nitinol still represents the material of choice in the biomedical field for its above-mentioned peculiar characteristics. More recently, SMPs have started to be considered as an interesting alternative. Indeed, as compared with SMAs, they are lightweight, compliant with many cost-effective manufacturing processes, can be combined with specific adjuvants to attain products with innovative properties and their shape recovery may be triggered by different external *stimuli*. The so-called multifunctional materials have aroused particular interest in that they show many attractive properties, such as biocompatibility, mechanical characteristics matching those of soft biological tissues, sterilizability and solubility and/or biodegradability, the latter being especially useful in case of products intended for temporary applications. Overall, the simple processability combined with the possibility of fine-tuning the mechanical properties and the actuation *stimulus* (*e.g.* activation temperature) have further boosted the interest in SMPs. The activation of the shape memory effect through heating, either direct or indirect, may lead to an undesired increase of temperature in the target area, potentially causing thermal damage of the surrounding tissues. In this respect, water-induced shape memory response could be highly advantageous. Indeed, water is always present in physiological environments - such as the bloodstream and the urinary bladder - and, acting as a plasticizer, may reduce the temperature required to trigger the targeted shape modifications. This is achieved when interaction with water causes an increase in the mobility of selected macromolecular chains, typically when dealing with polymers having hydrophobic and hydrophilic domains.

Several SMP-based systems intended for implantation have been described in the scientific literature, mainly for surgical and cardiovascular applications. This is the case with micrometric drug delivery carriers, self-tightening sutures, catheters, endovascular stents and clot removal systems.

**Fig. 7:**
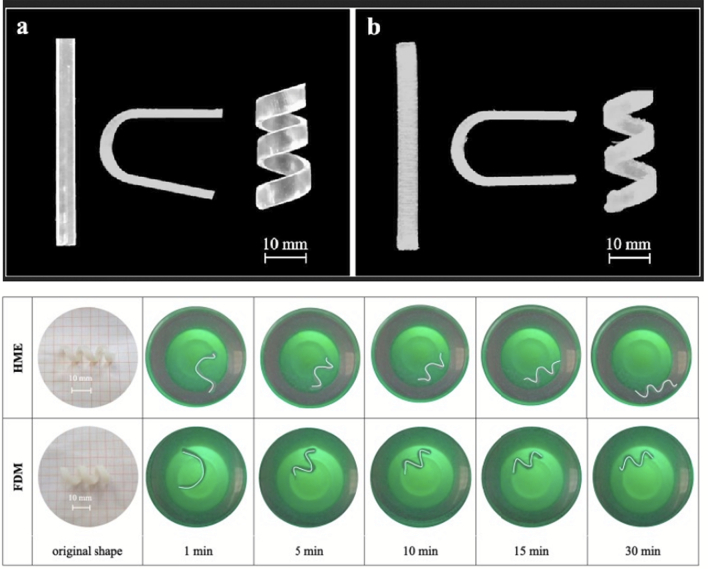
Photographs of originally I-, U- and helix-shaped intravesical specimens based on PVA obtained by (a) HME or (b) FDM and acquired on specimens having original helix shape, programmed to take on a temporary I-shape, during shape recovery experiments (bottom). Reprinted with permission from (Melocchi et al., 2019a).

As a further step forward and with the aim of circumventing invasive removal of exhausted devices, the use of water-soluble SMPs has been proposed for the development of indwelling systems for intra-organ release of drugs (Maroni et al., 2020; Melocchi et al., 2019a, Melocchi et al., 2019b). Particularly, poly(vinyl alcohol) (PVA) of pharmaceutical grade has been investigated based on preliminary data on its water-induced shape memory effect. Moreover, it is a thermoplastic polymer suitable for hot melt extrusion (HME) and fused deposition modeling (FDM) 3D printing, which allows for great versatility in terms of achievable shapes and sizes (Melocchi et al., 2018, Melocchi et al., 2020a, Melocchi et al., 2021a). Interestingly, the potential of FDM for the fabrication of personalized drug products has drawn special attention, and 3D printing coupled with the use of SMPs has been translated into 4D printing, the time frame during which the shape modifications takes place being the 4th dimension (Melocchi et al., 2020b, Melocchi et al., 2021b). Prototypes for intravesical delivery having rather simple original shapes (*i.e.* I-, U- and helix shapes) have been obtained by both the above-mentioned hot-processing techniques and manually deformed into differing temporary shapes (*i.e.* U- and I- shapes) (Fig. 7) (Melocchi et al., 2019a). Upon immersion in simulated urine fluid at body temperature, the samples show controlled release of the loaded drug tracer and the expected shape recovery effect. Feasibility of the proposed approach relying on 4D printing for the fabrication of retentive DDSs has therefore been demonstrated.

However, there are still open challenges, mainly involving the duration of release and mechanical properties of the system upon interaction with aqueous fluids.

## 6 Conclusions

Considering the current medical treatments of urinary bladder diseases and their prevalence in the population, development of advanced delivery systems conveying reduced though effective drug doses directly to the site of interest appears a priority in the pharmaceutical area. Direct and indirect savings as well as social benefits arising from use of intravesical delivery systems would especially be provided by devices that, once inserted into the bladder, may be retained within the organ and yield sustained release of the drug for the desired time frame, at least hours, requiring no other specific intervention. Indeed, this could limit the need for catheterization, reduce involvement of trained healthcare personnel and enhance the perceived quality of life for the patients. Furthermore, the decreased number of catheterizations could remarkably lower the incidence of secondary infections.

Given the growing interest in precision medicine, the possibility of personalizing the pharmacological therapy in terms of type as well as dose of drugs and release performance is also of utmost interest. Among the various formulation strategies described for bladder retention and delivery, systems based on externally-triggered expansion, elastic or smart materials have mainly been proposed, fabricated by different techniques. Particularly, the use of 3D and 4D printing, when dealing with shape-memory polymers, has been proved specially suited for fabrication of customizable therapeutic systems. Allowing patients to lead a regular daily life in spite of the disease conditions they have to face and, at the same time, receive a less invasive and more effective therapy, could result in an increase in the well-being of the treated subjects and in a greater adherence to the treatment, with further improvement of life expectancy.

## Declaration of Competing Interest

The authors report no conflicts of interest.
